# Complications during hospitalization and at 30 days in the intensive cardiac care unit for patients with ST-elevation versus non-ST-elevation acute coronary syndrome: A protocol for systematic review and meta analysis

**DOI:** 10.1097/MD.0000000000020655

**Published:** 2020-06-12

**Authors:** Qian Yang, Jinlong Du, Bing Wang

**Affiliations:** Department of Critical Care Medicine, Jingzhou Central Hospital, The Second Clinical Medical College, Yangtze University, Jingzhou, Hubei, P.R. China.

**Keywords:** complications, coronary care unit, intensive cardiac care unit, mortality, non-ST elevation acute coronary syndrome, re-infarction, ST elevation acute coronary syndrome

## Abstract

**Background:**

In this meta-analysis, we aimed to systematically compare the complications during hospitalization and at 30 days respectively, in intensive cardiac care unit (ICCU) for patients with ST elevation (STE) vs non-STE acute coronary syndrome (NSTE ACS).

**Methods:**

Electronic search databases including http://www.ClinicalTrials.gov, EMBASE, Cochrane Central, Google Scholar, Web of Science, and MEDLINE were searched for publications comparing complications observed in STE ACS vs NSTE ACS patients admitted in ICCU, intensive care unit (ICU) or coronary care unit (CCU). This is a meta-analysis and risk ratios (RR) with 95% confidence intervals (CI) were used to illustrate the data following analysis by the RevMan 5.3 software.

**Results:**

Six studies consisting of a total number of 25,604 participants (12,880 participants admitted due to STE ACS and 12,724 participants admitted due to NSTE ACS) were included. Our results showed that the total outcomes including severely abnormal electrocardiography (ECG) (RR: 1.48, 95% CI: 1.27–1.73; *P* = .00001) and mortality (RR: 1.83, 95% CI: 1.64–2.04; *P* = .00001) were significantly higher in patients with STE ACS. Re-infarction (RR: 0.86, 95% CI: 0.62–1.19; *P* = .37) and heart failure (RR: 1.04, 95% CI: 0.88–1.23; *P* = .62) were similarly manifested in those patients with ACS. However, the risk for recurrent angina was significantly higher with NSTE ACS (RR: 0.65, 95% CI: 0.46–0.92; *P* = .01).

**Conclusions:**

Patients with STE ACS were at a higher risk for in-hospital and 30 days mortality in this analysis. In hospital, severely abnormal ECG was also significantly higher in this category of patients compared to NSTE ACS. However, re-admission for heart failure and re-infarction was similar in both groups. Future studies should be able to confirm this hypothesis.

## 1 Introduction

Since its existence, the intensive care unit (ICU) has been the main section of the hospital to handle severely ill patients with several co-morbidities and who would require urgent assistance and monitoring. From the time, they were set up, the intensive cardiac care units (ICCU) and the coronary care units (CCU) have been the main sections of specialized centers to handle and admit complicated and severely unstable patients with acute coronary syndrome (ACS).^[1,2]^

Data on patients admitted to the ICCU or CCU have not been easy to obtain. The BLITZ-4 Qualita comprising of CCUs across Italy which was launched by the Italian Association of Hospital Cardiologists (Associazione Nazionale Medici Cardiologi Ospedalieri) aimed at collecting data of the patients with ST elevation (STE) and non-ST elevation (NSTE) ACS.^[3]^ In addition, the Italian Association of Hospital Cardiologists (ANMCO) and the Italian Health Institute (IHI) were involved to form the Italian network on ACS outcome (IN-ACS Outcome) study to further collect data based on outcomes of such patients admitted to the CCU.^[4]^ The French prospective study, Unite de Soins Intensifs Coronaires (USIC) was also set up to follow-up on such ACS patients after discharge from CCU.^[5]^ However, the complications in STE and NSTE ACS patients who were admitted in the ICCU or CCU were never systematically compared.

In this meta-analysis, we aimed to systematically compare the complications during hospitalization and at 30 days respectively, in ICCU for patients with STE vs NSTE ACS.

## 2 Materials and methods

### 2.1 Searched databases and searched strategies

Electronic search databases: http://www.ClinicalTrials.gov, EMBASE, Cochrane Central, Google Scholar, Web of Science, and MEDLINE were searched for publications comparing complications observed in STE ACS vs NSTE ACS patients admitted in the ICCU, ICU, or CCU.

The following searched terms/phrases/texts were used to find publications:

1. ICU and ACS;
2. ICU and ACS;
3. ICU and ACS;
4. ICU and ACS;
5. ICU and myocardial infarction;
6. ICU and STE myocardial infarction;
7. ICU and STE ACS;
8. ICU and percutaneous coronary intervention;
9. ICU and coronary angioplasty.

The term “intensive care unit” was also replaced by “intensive cardiac care unit,” “coronary care unit,” and “cardiac care unit.”

This search was restricted only to articles which were published in English language.

### 2.2 Major criteria for inclusion

Major criteria for inclusion were:

1. Studies comparing the complications of STE vs NSTE ACS patients admitted to the ICCU;
2. Studies that had an in-hospital follow-up or a follow-up time period of 30 days.

### 2.3 Major criteria for exclusion

The major criteria for exclusion were:

1. Literature reviews, meta-analyses, case reports and correspondence;
2. Studies that were published in a different language apart from English;
3. Studies that did not compare the complications of STE vs NSTE ACS patients admitted to the ICCU;
4. Duplication of studies.

### 2.4 Outcomes which were reported

Table 1 lists the outcomes which were reported during hospitalization and at 30 days following ICCU admission.

**Table 1:**
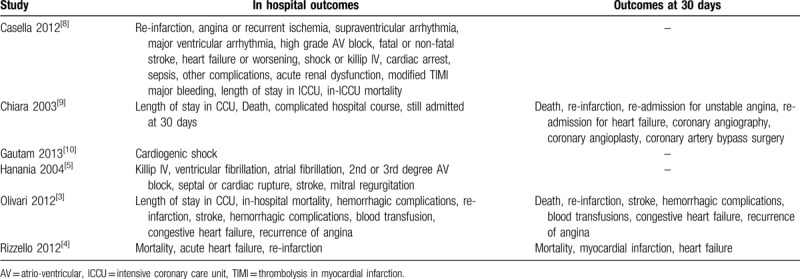
Outcomes (in hospital and at 30 days).

The endpoints which were assessed included:

1. Re-infarction;
2. Recurrent angina;
3. Heart failure;
4. Stroke;
5. Severely abnormal electrocardiography (ECG) including atrial and ventricular fibrillations, supraventricular tachycardia and atrio-ventricular blocks;
6. Mortality.

### 2.5 Data extraction and quality assessment

All the authors were independently involved in the data extraction process. First of all the complications which were reported in the hospital and at 30 days in each study were carefully extracted followed by the total number of participants in each group, the participants’ enrollment time period, the type of study, the co-morbidities of the participants, the age, and gender, the total number of events occurring in each subgroup were carefully extracted.

Disagreement concerning data extraction was discussed among the authors and finally solved by consensus.

The Newcastle Ottawa Scale (NOS),^[6]^ a tool to assess the methodological quality of the studies was used during the assessment of the studies, and grades were allotted: Grade A (low risk bias), Grade B (moderate risk of bias), and Grade C (high risk of bias).

### 2.6 Statistical analysis

This is a meta-analysis and risk ratios (RR) with 95% confidence intervals (CI) were used to illustrate the data following analysis by the RevMan 5.3 software. The *Q* statistic test was used to assess heterogeneity. A significance level *P* ≤ .05 was set implying that any subgroup analysis with *P* value less or equal to .05 was considered statistically significant. Heterogeneity was also assessed with the *I*^2^ statistic test and heterogeneity increased with an increasing *I*^2^ value. In addition, if the heterogeneity was high, a random statistical model effect was used, whereas a fixed effect model was used if the heterogeneity was low.

Sensitivity analysis was also carried out by excluding each of the study one by one by turn, and any significant change in the result was observed.

Funnel plots were generated from the RevMan software and they were used to visually assess publication bias.

### 2.7 Ethical approval

This study does not include experiments that were carried out on humans or animals by any of the authors. Therefore, an ethical or a board review approval was not required.

## 3 Results

### 3.1 Search outcomes

One thousand three hundred fourteen (1314) publications were obtained. An initial evaluation was carried out to eliminate the less relevant studies. At first, 986 publications were eliminated. Three hundred twenty-eight (328) full text articles were assessed for eligibility. A further 264 full text articles were eliminated for irrelevant contents.

Among the 64 remaining articles, 3 were eliminated since they were published in a different language, 5 were eliminated since they were literature reviews, 2 were letters of correspondence, 7 were case studies, 8 did not report the required outcomes, and 33 were duplicated studies. This selection process was based on the PRISMA guideline.^[7]^

**Figure 1:**
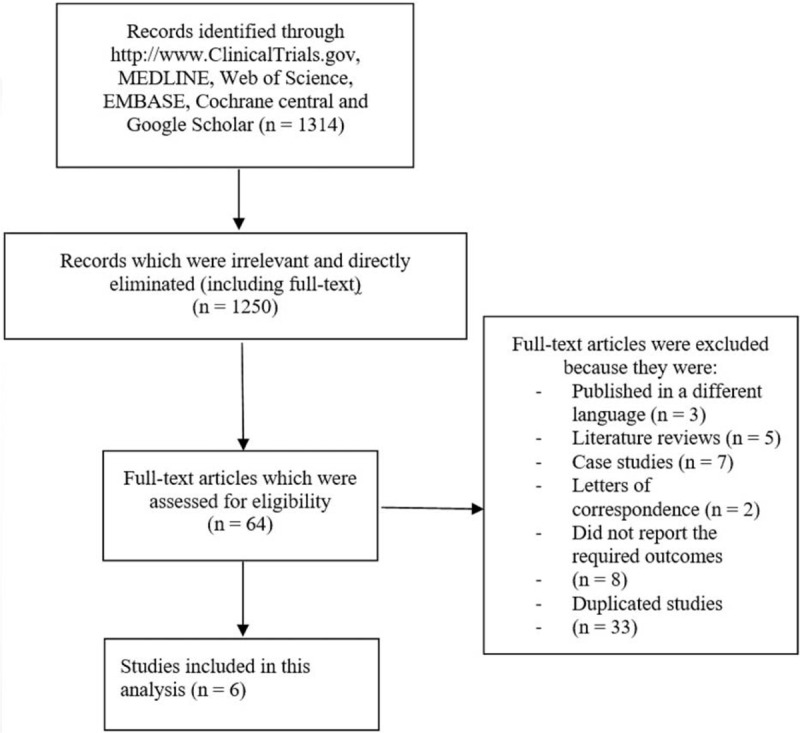
Flow diagram for the study selection.

Finally, only 6 articles^[3–5,8–10]^ were selected for this analysis (Fig. 1).

### 3.2 Main features and baseline characteristics of the selected studies

The main features of the included studies have been listed in Table 2. All the studies were observational registries or cohorts. The patients were admitted to either the ICU, ICCU, or the CCU. The time period for enrollment of these participants ranged from the years 2000 to 2010. The six studies consisted of a total number of 25,604 participants (12,880 participants admitted due to STE ACS and 12,724 participants admitted due to NSTE ACS). Based on the NOS assessment, a grade B was allotted to the studies indicating a moderate risk of bias.

**Table 2:**
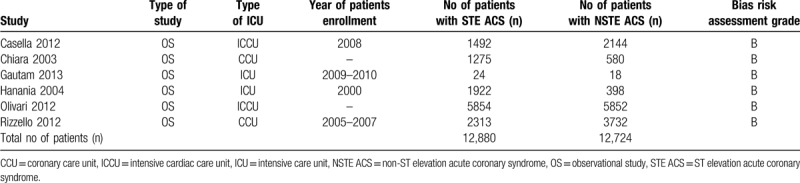
Main features of the studies.

Table 3 lists the baseline features of the studies whereas Table 4 lists some of the major features of the participants. Majority of the participants admitted to ICCU were male patients with a mean age ranging from 64.5 to 71.0 years. Co-morbidities including diabetes mellitus, hypertension and atrial fibrillation were also given in Table 3. The types of coronary diseases, the percentage of participants with renal dysfunction, prior myocardial infarction, prior revascularization, and the percentage of participants with Killip score 1 to 4 have been listed in Table 4.

**Table 3:**
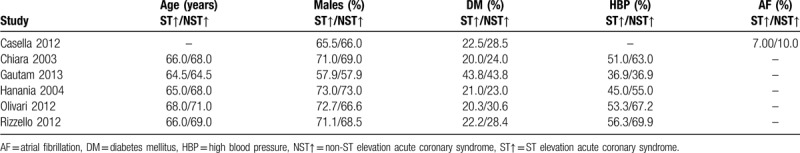
Baseline features of the studies.

**Table 4:**
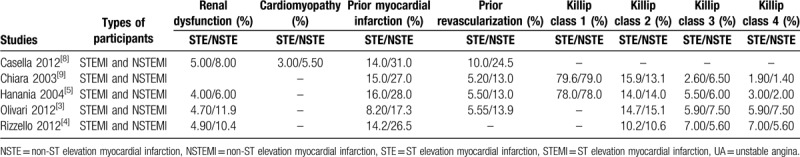
Types of coronary artery disease and some basic features.

### 3.3 Results of this analysis

**Figure 2:**
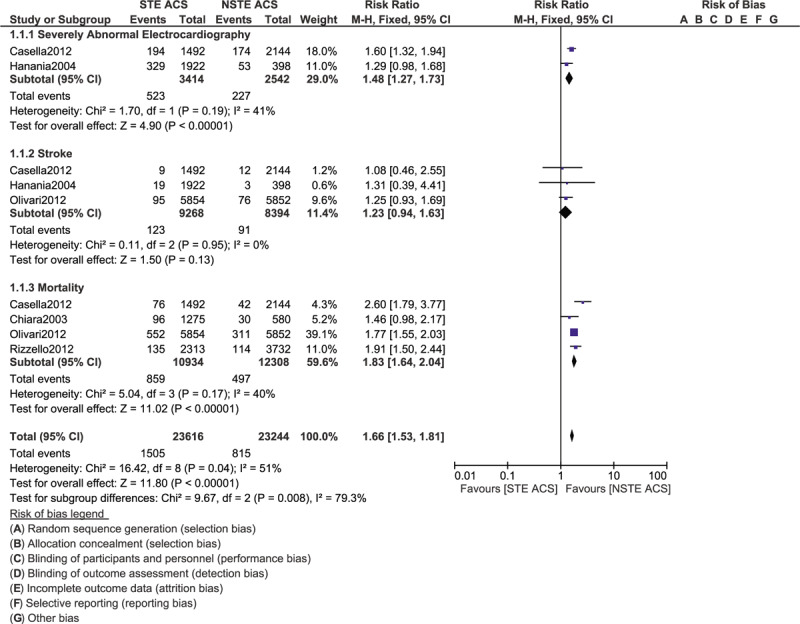
Comparing the overall complication risks in patients with STE and NSTE ACS admitted to the Intensive cardiac care unit (Part I).

**Figure 3:**
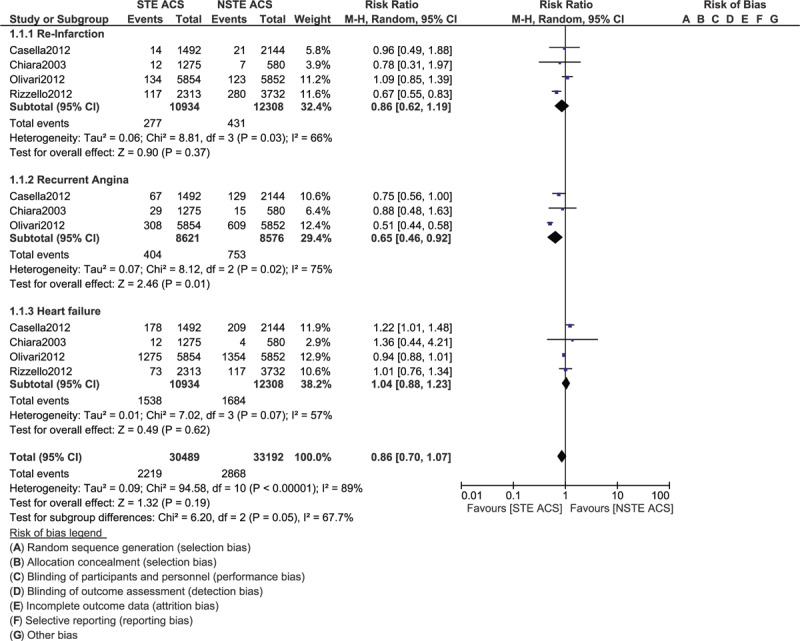
Comparing the overall complication risks in patients with STE and NSTE ACS admitted to the Intensive cardiac care unit (Part II).

Our analysis showed that the total outcomes including severely abnormal ECG (RR: 1.48, 95% CI: 1.27–1.73; *P* = .00001) and mortality (RR: 1.83, 95% CI: 1.64–2.04; *P* = .00001) were significantly higher in patients with STE ACS as shown in Figure 2. Stroke was not significantly different (RR: 1.23, 95% CI: 0.94–1.63; *P* = .13). Re-infarction (RR: 0.86, 95% CI: 0.62–1.19; *P* = .37) and heart failure (RR: 1.04, 95% CI: 0.88–1.23; *P* = .62) were also similarly manifested in those patients with ACS as shown in Figure 3. However, the risk for recurrent angina was significantly higher with NSTE ACS (RR: 0.65, 95% CI: 0.46–0.92; *P* = .01) as shown in Figure 3.

The outcomes were also separately assessed based on the in hospital follow-up and follow-up at 30 days, respectively.

**Figure 4:**
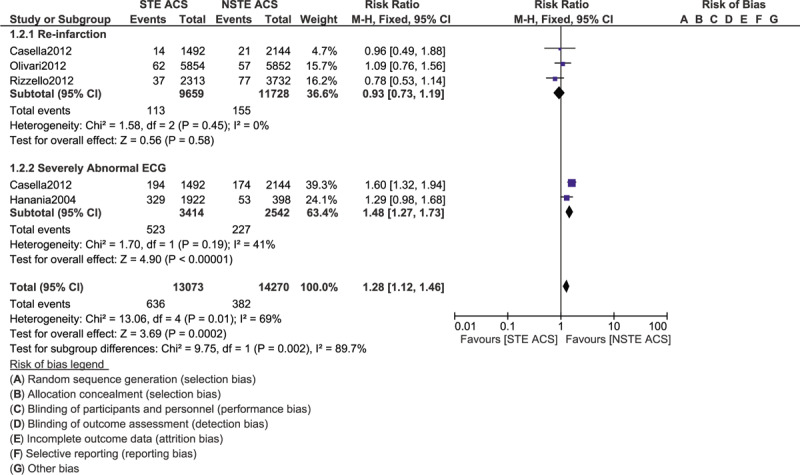
Comparing the in-hospital complication risks in patients with STE and NSTE ACS admitted to the Intensive cardiac care unit (Part I).

**Figure 5:**
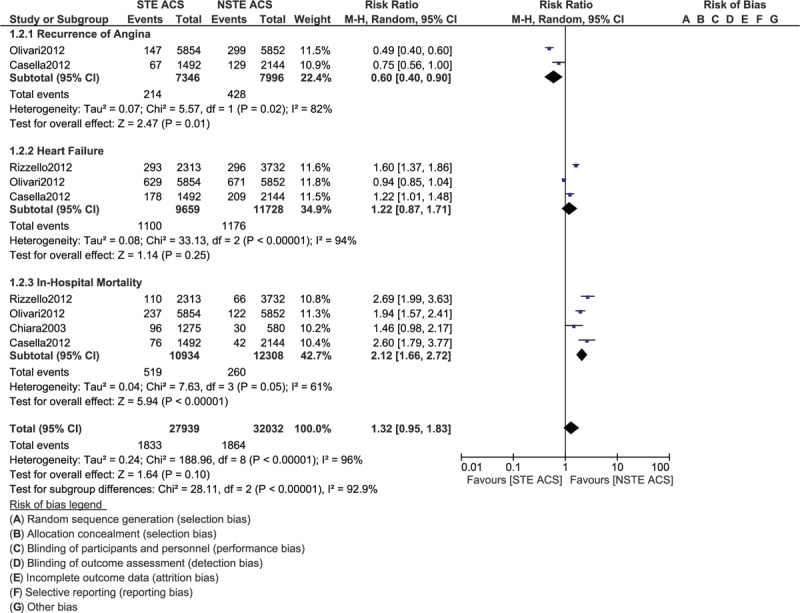
Comparing the in-hospital complication risks in patients with STE and NSTE ACS admitted to the Intensive cardiac care unit (Part II).

During the in-hospital follow-up time period, severely abnormal ECG (RR: 1.48, 95% CI: 1.27–1.73; *P* = .00001) was significantly higher in patients with STE ACS as shown in Figure 4. In-hospital mortality was also significantly higher with STE ACS (RR: 2.12, 95% CI: 1.66–2.72; *P* = .00001) as shown in Figure 5. However, re-infarction and heart failure were not significantly different with (RR: 0.93, 95% CI: 0.73–1.19; *P* = .58) and (RR: 1.22, 95% CI: 0.87–1.71; *P* = .25), respectively during this in-hospital follow-up time period. Recurrence of angina was also significantly increased in patients with NSTE ACS (RR: 0.60, 95% CI: 0.40–0.90; *P* = .01).

**Figure 6:**
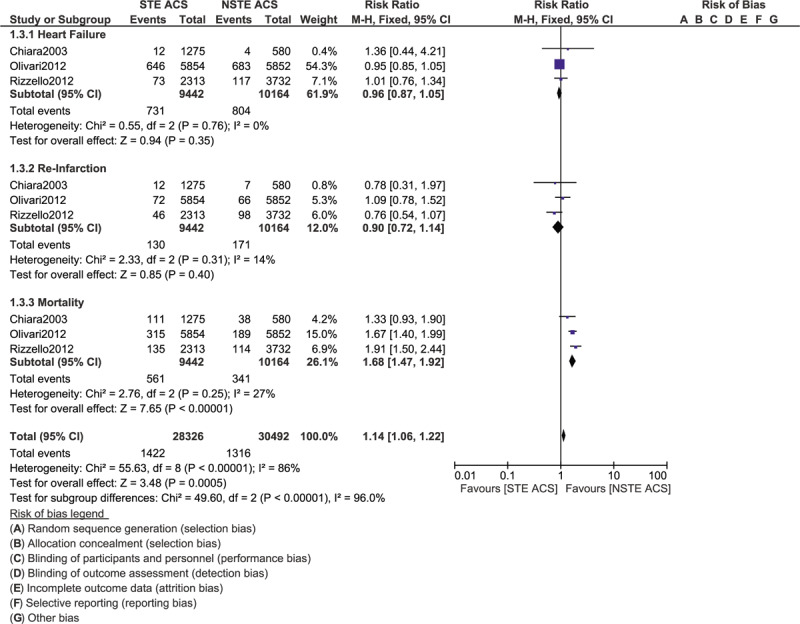
Comparing the complication risks at 30 days in patients with STE and NSTE ACS admitted to the Intensive cardiac care unit.

At 30 days, heart failure (RR: 0.96, 95% CI: 0.87–1.05; *P* = .35) and re-infarction (RR: 0.90, 95% CI: 0.72–1.14; *P* = .40) were still similarly manifested in those patients with STE and NSTE ACS as shown in Figure 6. However, mortality was significantly higher with STE ACS (RR: 1.68, 95% CI: 1.47–1.92; *P* = .00001) as shown in Figure 6.

A summarized version reflecting the results of this analysis has been given in Table 5.

**Table 5:**
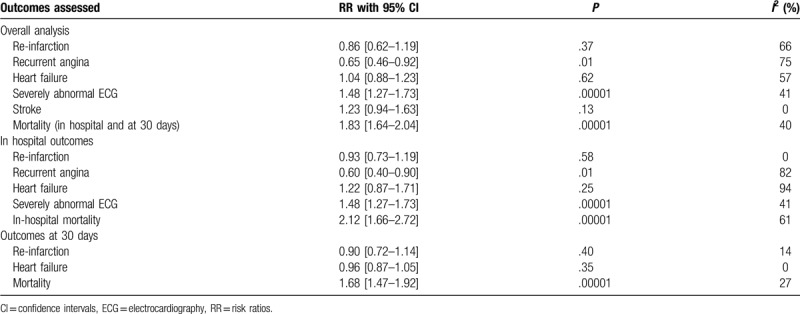
Results of this analysis.

### 3.4 Sensitivity analysis and publication bias

**Figure 7:**
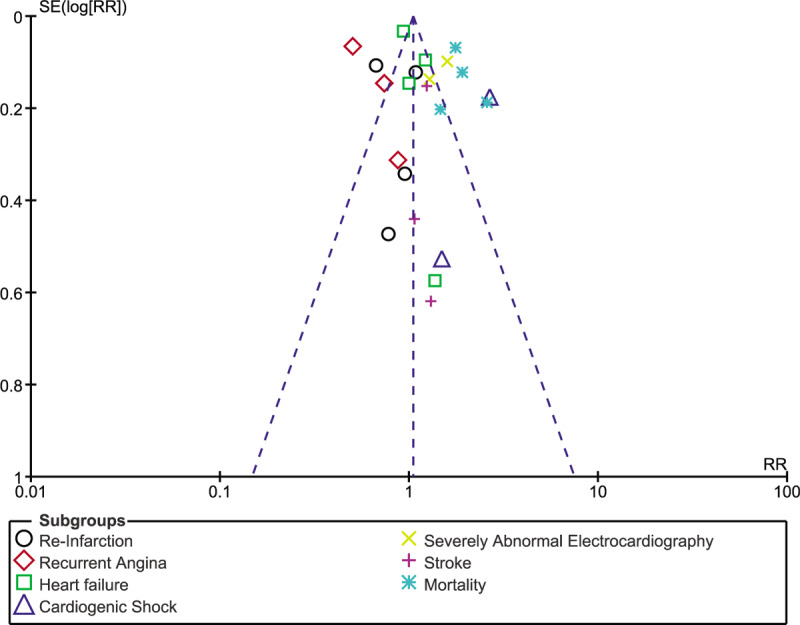
Funnel plot representing publication bias for studies involved to assess the overall outcomes.

**Figure 8:**
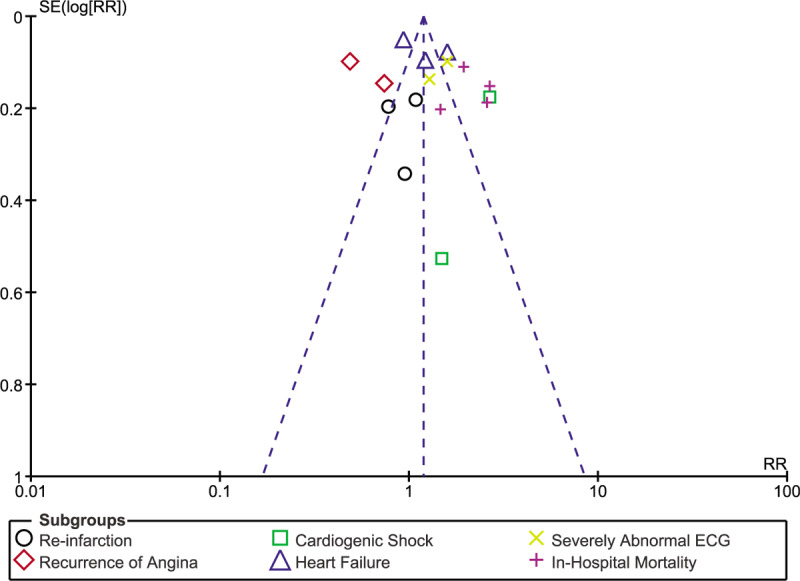
Funnel plot representing publication bias for studies involved to assess outcomes during in-hospital follow-up.

**Figure 9:**
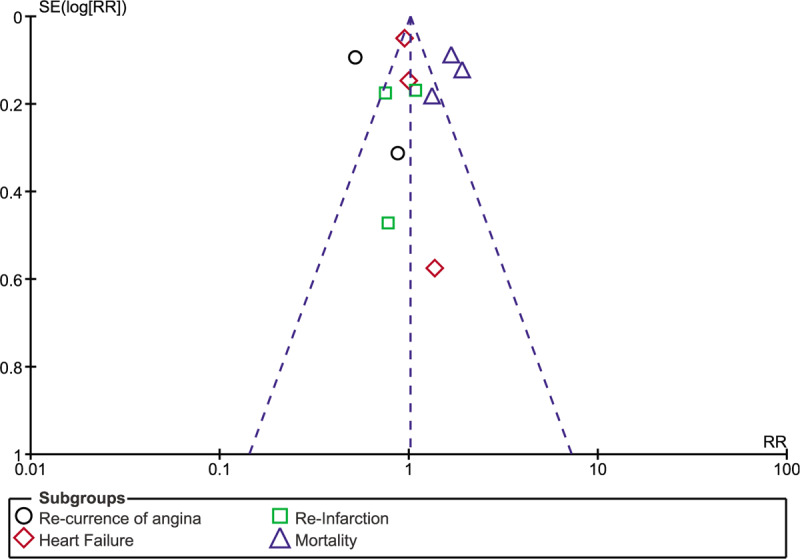
Funnel plot representing publication bias for studies involved to assess outcomes at 30 days follow-up.

Consistency in results was observed throughout following sensitivity analysis. There was only low evidence of publication bias as shown in Figures 7–9 which visually demonstrated assessment of publication bias using studies which were involved for overall outcome, in-hospital outcomes and outcomes observed at 30 days, respectively.

## 4 Discussion

In this present analysis, severely abnormal ECG (atrial and ventricular fibrillations, ventricular tachycardia, and atrio-ventricular blocks) and mortality were significantly higher in ICCU patients with STE ACS as compared to NSTE ACS. This result mainly reflected the complications observed during the hospitalization time period. However, even at 30 days follow-up, the risk of mortality was still significantly higher in patients with STE ACS. In contrast, our analysis showed that recurrent angina was significantly higher in the NSTE ACS patients when compared to the STE ACS participants. However, re-infarction and heart failure were similar during the in-hospital and follow-up at 30 days in both groups.

An analysis involving 10, 983 participants with NSTE ACS from 5 Italian nationwide registries (2001–2010) consisting of patients admitted to the CCU, showed that complications were less and there was a reduction in the 30 day mortality among patients with NSTE ACS. This was also the case in our current meta-analysis showing a significantly higher risk of mortality to be associated with the STE ACS patients.^[11]^

In this present analysis, the mean age of the participants varied from 64.5 to 71.0 years. Other studies have shown this age factor to significantly contribute to the prognosis of ACS. Old age is a major predictor of mortality in patients with ACS.^[12]^ The Euroheart ACS survey^[13]^ which involved academic and non-academic hospitals with or without catheterization laboratories, as well as with or without cardiac surgery facilities, and enrolling patients from the year 2000 to 2001, from 25 different countries, it was found that the rate of STE ACS was less in elder patients, but, however, in-hospital mortality was more likely in the subgroup of patients with STE ACS as demonstrated in this present analysis.

In this analysis, a significantly higher rate of recurrent unstable angina was observed in the NSTEMI group among all those patients who were admitted in CCU. Possible explanations could be the presence of collateral vessels, there might be a flow limiting condition for example a stable plaque, a small coronary embolism or vasospasm which might not be severe enough to cause an elevation in cardiac biomarkers.^[14]^ Conditions such as hypertension, tachycardia and cardio-toxic drugs might also be reasons which could induce an angina.^[15]^

In this present analysis, data were insufficient to show an analysis for cardiogenic shock in patients with STE ACS. In a CCU in the National Hospital of Sri Lanka,^[16]^ where 139 consecutive patients were admitted with STE ACS, mortality in 4 patients were due to cardiogenic shock, indicating that this complication is also quite common in STE ACS patients admitted to ICCU. However, future studies should assess more of such complications in patients with ACS admitted to the ICCU.

Even though percutaneous coronary intervention is best suited for less complicated coronary artery disease, recent studies have shown that in more complicated coronary diseases, especially with involvement of the left main coronary artery, both percutaneous coronary intervention and coronary artery bypass surgery have proven to be equally effective, with no significant difference in complications.^[17]^ It should also be noted that in patients with acute myocardial infarction, thrombectomy could result in immediately improved angiographic results and better clinical outcomes when compared to conventional percutaneous coronary intervention.^[18]^ At last, it should be understood that managing ACS in an ICU setting requires professional skills and intense knowledge since complications which arise might not be easy to manage.

## 5 Limitations

Limitations of this analysis were as followed: The total number of studies which were included in this meta-analysis was less, but we could not improve this limitation since there was no other studies that satisfied the inclusion and exclusion criteria of this analysis. Secondly, due to the limited number of studies and due to the fact that all the endpoints were not reported in all the studies, each subgroup analysis assessing respective outcomes could not include all the studies. Moreover, confounding variables might have contributed to bias in this analysis. Also, during analysis of heart failure, we also included one study reporting re-hospitalization for heart failure during a follow-up time period of 1 year in the 30 day category. This might to a minor extent, affect the result for heart failure. In addition, there was one study which included a very minor number of participants; however, since the study satisfied the inclusion and exclusion criteria of this analysis, we could not have ignored it.

## 6 Conclusions

Patients with STE ACS were at a higher risk for in-hospital and 30 days mortality in this analysis. In hospital, severely abnormal ECG was also significantly higher in this category of patients compared to NSTE ACS. However, re-admission for heart failure and re-infarction was similar in both groups. Future studies should be able to confirm this hypothesis.

## Author contributions

The authors Qian Yang, Jinlong Du, and Bing Wang were responsible for the conception and design, acquisition of data, analysis and interpretation of data, drafting the initial manuscript and revising it critically for important intellectual content. The first co-authors Qian Yang and Jinlong Du wrote this manuscript. All the authors agreed to and approved the manuscript as it is.

**Conceptualization:** Qian Yang, Jinlong Du, Bing Wang.

**Data curation:** Qian Yang, Jinlong Du, Bing Wang.

**Formal analysis:** Qian Yang, Jinlong Du, Bing Wang.

**Funding acquisition:** Qian Yang, Jinlong Du, Bing Wang.

**Investigation:** Qian Yang, Jinlong Du, Bing Wang.

**Methodology:** Qian Yang, Jinlong Du, Bing Wang.

**Project administration:** Qian Yang, Jinlong Du, Bing Wang.

**Resources:** Qian Yang, Jinlong Du, Bing Wang.

**Software:** Qian Yang, Jinlong Du, Bing Wang.

**Supervision:** Qian Yang, Jinlong Du, Bing Wang.

**Validation:** Qian Yang, Jinlong Du, Bing Wang.

**Visualization:** Qian Yang, Jinlong Du, Bing Wang.

**Writing – original draft:** Qian Yang, Jinlong Du.

**Writing – review & editing:** Qian Yang, Jinlong Du.

## References

[1] Le May M van Diepen S Liszkowski M From coronary care units to cardiac intensive care units: recommendations for organizational, staffing, and educational transformation. Can J Cardiol 2016;32:1204–13. 26968391 10.1016/j.cjca.2015.11.021

[2] Valentin A Ferdinande P ESICM Working Group on Quality Improvement. Recommendations on basic requirements for intensive care units: structural and organizational aspects. Intensive Care Med 2011;37:1575–87. 21918847 10.1007/s00134-011-2300-7

[3] Olivari Z Steffenino G Savonitto S The management of acute myocardial infarction in the cardiological intensive care units in Italy: the ’BLITZ 4 Qualità’ campaign for performance measurement and quality improvement. Eur Heart J Acute Cardiovasc Care 2012;1:143–52. 24062902 10.1177/2048872612450520 PMC3760526

[4] Rizzello V Lucci D Maggioni AP IN-ACS Outcome Investigators. Clinical epidemiology, management and outcome of acute coronary syndromes in the Italian network on acute coronary syndromes (IN-ACS Outcome study). Acute Card Care 2012;14:71–80. 22452295 10.3109/17482941.2012.655296

[5] Hanania G Cambou JP Guéret P Management and in-hospital outcome of patients with acute myocardial infarction admitted to intensive care units at the turn of the century: results from the French nationwide USIC 2000 registry. Heart 2004;90:1404–10. 15547013 10.1136/hrt.2003.025460 PMC1768566

[6] Stang A Critical evaluation of the Newcastle-Ottawa scale for the assessment of the quality of nonrandomized studies in meta-analyses. Eur J Epidemiol 2010;25:603–5. 20652370 10.1007/s10654-010-9491-z

[7] Liberati A Altman DG Tetzlaff J The PRISMA statement for reporting systematic reviews and meta-analyses of studies that evaluate healthcare interventions: explanation and elaboration. BMJ 2009;339:b2700. 19622552 10.1136/bmj.b2700 PMC2714672

[8] Casella G Scorcu G Cassin M Elderly patients with acute coronary syndromes admitted to Italian intensive cardiac care units: a Blitz-3 Registry sub-analysis. J Cardiovasc Med (Hagerstown) 2012;13:165–74. 22306786 10.2459/JCM.0b013e3283515be3

[9] Di Chiara A Chiarella F Savonitto S Epidemiology of acute myocardial infarction in the Italian CCU network: the BLITZ study. Eur Heart J 2003;24:1616–29. 14499224 10.1016/s0195-668x(03)00278-1

[10] Gautam MP Sogunuru G Subramanyam G Acute coronary syndrome in an intensive care unit of a tertiary care centre: the spectrum and coronary risk factors. JNMA J Nepal Med Assoc 2013;52:316–21. 24362653

[11] De Luca L Olivari Z Bolognese L A decade of changes in clinical characteristics and management of elderly patients with non-ST elevation myocardial infarction admitted in Italian cardiac care units. Open Heart 2014;1:e000148. 25525506 10.1136/openhrt-2014-000148 PMC4267110

[12] Rubinstein R Matetzky S Beigel R Trends in management and outcome of acute coronary syndrome in women ≥80 years versus those <80 years in Israel from 2000-2016. Int J Cardiol 2019;281:22–7. 30709558 10.1016/j.ijcard.2019.01.076

[13] Rosengren A Wallentin L Simoons M Age, clinical presentation, and outcome of acute coronary syndromes in the Euroheart acute coronary syndrome survey. Eur Heart J 2006;27:789–95. 16464911 10.1093/eurheartj/ehi774

[14] Basit H Malik A Huecker MR Non ST segment elevation (NSTEMI) myocardial infarction. 30020600

[15] Kamińska J Koper OM Siedlecka-Czykier E The utility of inflammation and platelet biomarkers in patients with acute coronary syndromes. Saudi J Biol Sci 2018;25:1263–71. 30505168 10.1016/j.sjbs.2016.10.015 PMC6252018

[16] Rahuman MB Jayawardana JB Francis GR Outcome of early coronary intervention for acute ST elevation myocardial infarction in a tertiary care cardiac centre in Sri Lanka. Ceylon Med J 2016;61:26–31. 27031976 10.4038/cmj.v61i1.8258

[17] De Rosa S Polimeni A Sabatino J Long-term outcomes of coronary artery bypass grafting versus stent-PCI for unprotected left main disease: a meta-analysis. BMC Cardiovasc Disord 2017;17:240. 28877676 10.1186/s12872-017-0664-5 PMC5588710

[18] De Rosa S Cirillo P De Luca G Rheolytic thrombectomy during percutaneous coronary intervention improves long-term outcome in high-risk patients with acute myocardial infarction. J Interv Cardiol 2007;20:292–8. 17680859 10.1111/j.1540-8183.2007.00271.x

